# Drinking Water Disinfection Using Nutritional Level Zinc Assisted with Electric Field Treatment

**DOI:** 10.1021/acs.est.6c03361

**Published:** 2026-04-29

**Authors:** Wei Wang, Mourin Jarin, Farshid Khan, Feiyang Mo, Shuai Wang, Kaiqin Bian, Anuja Tripathi, Ameet J Pinto, Xing Xie

**Affiliations:** School of Civil and Environmental Engineering, 1372 Georgia Institute of Technology, Atlanta, Georgia 30332, United States

**Keywords:** chlorine-free disinfection, electric field treatment, zinc fortification, microbial inactivation, electroporation

## Abstract

Next-generation drinking water treatment should not only ensure effective disinfection without generating harmful byproducts but also contribute to human health. Herein, we report the first demonstration of a combined electric field treatment and zinc (EFT-Zn) process that achieves water disinfection at Zn concentrations within safe and nutritionally beneficial limits, providing a previously unexplored approach for applying Zn in drinking water disinfection. Using a lab-on-a-chip device, we directly visualized and quantified the synergistic effect between electric field exposure and Zn < 5 mg/L for microbial inactivation. This synergistic mechanism was further validated in a bench-scale continuous-flow reactor, achieving ∼3-log inactivation of *Staphylococcus epidermidis* and *Escherichia coli* at 1.5 V and 3 mg/L Zn. A scaled-up system was subsequently developed and demonstrated effective disinfection of natural surface waters at a practical flow rate of 420 mL/min. Collectively, these findings establish EFT-Zn as a scalable, efficient, and health-promoting water disinfection strategy that integrates both microbial safety with micronutrient fortification, providing a foundation for next-generation sustainable drinking water technologies.

## Graphical abstract

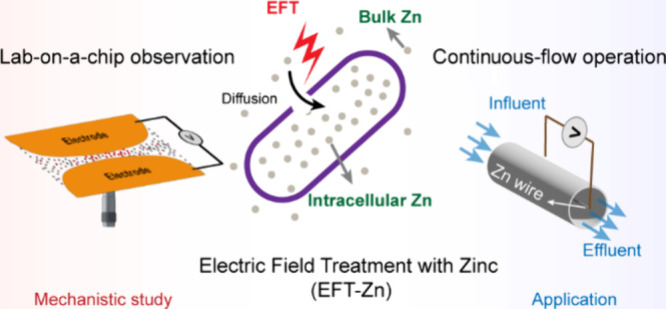

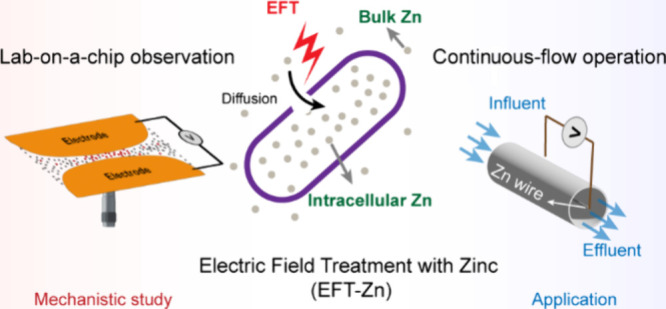

## Introduction

Zinc (Zn) is an essential trace element that plays critical roles in many physiological processes, such as enzyme catalysis, immune regulation, DNA synthesis, and cell signaling.^1–3^ Dietary sources such as meat, grains, and seafood are the primary contributors for Zn intake. However, more than 2 billion people worldwide are estimated to suffer from Zn deficiency,^4,5^ which can cause diarrhea, impaired immunity, slow growth, and increased susceptibility to infections.^6^ In such cases, additional Zn supplementation is often required to maintain normal physiological functions,^7,8^ and more than 30% of adults in the United States reported using Zn supplements between 1999 and 2014.^9^ Drinking water fortification has emerged as a practical and low-cost strategy to improve mineral intake, particularly in regions with limited dietary diversity.^10,11^ Zn-fortified water has been reported to reduce childhood infections such as colds, diarrhea, and abdominal pain.^12,13^ One method for enabling Zn fortification involves incorporating a glassy zinc phosphate plate into household water units to release zinc into the water.^12^ However, the dissolution rate of this Zn plate is highly sensitive to water temperature, hardness and other water quality factors, making it challenging to maintain consistent Zn concentrations.

Beyond its nutritional role, Zn also exhibits inherent antimicrobial properties,^14,15^ making it a promising candidate for water disinfection. Nevertheless, the minimum inhibitory and bactericidal concentrations of Zn ions against most bacteria are typically in the tens to hundreds of milligrams per liter range.^16–18^ These levels are far above drinking water guidelines, including the United States Environmental Protection Agency (USEPA) secondary maximum contaminant level of 5 mg/L and the World Health Organization (WHO) aesthetic guideline of <3 mg/L. The biocidal mechanism of Zn is primarily attributed to excessive cellular uptake and intracellular accumulation, followed by transformation into insoluble nanocomposites that disrupt cellular structures and metabolic functions, and the interference in the uptake of other essential trace metal ions.^15,18^ However, at the much lower Zn concentrations permitted in drinking water, Zn diffusion across the membrane and subsequent intracellular accumulation are insufficient to induce effective bacterial inactivation. We therefore hypothesize that if we apply strategies to enhance Zn ion transport into microbial cells and promote intracellular accumulation, we can achieve microbial inactivation at safe bulk Zn concentrations, providing simultaneous water disinfection and Zn supplementation at levels beneficial to human health.

Here, we introduce a new drinking water disinfection strategy that combines electric field treatment with Zn (EFT-Zn) to achieve dual benefits of efficient microbial inactivation and safe Zn fortification. EFT is an emerging physical treatment approach that inactivates microorganisms through membrane disruption.^19–21^ When cells are exposed to an external electric field, the induced transmembrane potential compresses the lipid bilayer, initiates ion transport channels, and ultimately enables the formation of transient or permanent pores.^22,23^ These effects significantly increase membrane permeability and facilitate the transport of small molecules and ions across the membrane.^24^ Previous studies have demonstrated that EFT can accelerate the diffusion of disinfectants such as chlorine and ozone, leading to improved inactivation efficiency with lower chemical dosage.^25–27^ Moreover, synergistic effects between EFT and antimicrobial metals like Cu and Ag have been observed where the applied electric field promotes metal ion uptake and enhances their antibacterial activity.^28,29^ Building on these findings, we hypothesize that a similar synergistic effect could exist between EFT and Zn, where the electric field facilitates Zn ion transport into microbial cells, resulting in superior inactivation performance compared to either treatment alone. We further hypothesize that this synergy could enable efficient and safe disinfection at Zn concentrations well within drinking water quality standards. However, to date, no prior study has systematically investigated the interaction between EFT and Zn, nor explored its feasibility for drinking water treatment.

To address this knowledge gap, we integrated microscale lab-on-a-chip (LOAC) analysis with macroscale continuous-flow disinfection experiments to systematically investigate the interaction between EFT and Zn for water disinfection at Zn concentrations within water quality standards and beneficial to health. We first employed a LOAC platform capable of generating well-controlled electric field gradients to visualize bacterial inactivation and quantify the synergistic effects of EFT and Zn under varying conditions. Based on the mechanistic insights obtained from the microscale study, a bench-scale EFT-Zn reactor was constructed to assess disinfection performance in continuous-flow conditions and to determine the key operational parameters. Finally, a scaled-up EFT-Zn system was developed and tested using both synthetic and natural surface waters to assess its scalability and real-word feasibility. Overall, this study reports a novel and scalable water disinfection strategy that not only ensures microbial safety, but also provides Zn fortification within regulatory limits, advancing the concept of safe and health-promoting drinking water treatment.

## Experimental Section

### LOAC Device Setup

The LOAC device was fabricated following previously reported methods with minor modifications.^28^ Briefly, gold electrodes with curved structure were patterned onto a glass wafer using standard photolithography and lift-off techniques. The curved electrode geometry was designed to produce a linear gradient of electric field strengths along the *x*-axis. The chip is 440 μm in length and 235 μm in width, with the narrowest electrode gap of 20 μm. For operando investigation, *Staphylococcus epidermidis* (ATCC 12228) was immobilized on the chip surface as a model bacterial strain and the detailed process is provided in the Supporting Information. The chip was inverted and mounted on a Zeiss Axio Observer 7 fluorescence microscope for the following treatment and all imaging experiments.

### Electric Field Treatment on the LOAC Device

In all EFT-based experiments on the LOAC device, electric pulses were applied using an Avtech AV-1010-B high-speed pulse generator, controlled by a waveform generator (Keysight 33509B). Operational parameters were set to a 70 V square-wave voltage, 500 ns pulse width, and 500 μs period. The total effective exposure time was maintained at 20 ms. These parameters were selected based on prior optimization to ensure effective inactivation while minimizing the generation of reactive oxygen species (ROS), bubble formation, and thermal effects.^28,30^ For EFT-Zn treatment, an aliquot of 100 μL zinc nitrate (Zn­(NO_3_)_2_) solution with Zn concentrations between 1 and 5 mg/L was added into the chip and the same electrical parameters were applied.

### Microscope Image Acquisition and Processing

Fluorescence and differential interference contrast (DIC) images were acquired using a Zeiss Axio Observer 7 fluorescence microscope equipped with a CCD camera. Inactivated cells were characterized by using a cell impermeable fluorescent dye, propidium iodide (PI). To ensure sufficient Zn exposure and avoid interference from transient electroporation pores, samples were incubated for 2 h after EFT and Zn treatment before PI staining and imaging. Image processing and quantitative analysis were performed using MATLAB (MathWorks 2023a), and the details are provided in the Supporting Information. Briefly, a 440 μm × 20 μm region was cropped from each image and divided into 120 vertical segments. Cell counts were obtained using binary color thresholding. Inactivation efficiency was calculated as the ratio of PI-labeled cells to total cells identified in the DIC channel. For each experiment, up to 30 repeat channels were analyzed, and data were aggregated from all usable channels to compute inactivation percentages along the *x*-axis.

### Bacteria Inactivation Experiments Using a Bench-Scale EFT-Zn Reactor

A bench-scale coaxial EFT-Zn device was constructed following previous established designs with minor modifications.^31,32^ Briefly, a stainless-steel cylindrical tube was used as outer cathode and mounted between two poly­(vinyl chloride) (PVC) end blocks with plugs on both ends and inlet and outlet ports. A zinc wire with a diameter of 355 μm was firmly suspended within the cylinder, serving as the center anode. This configuration enables a locally enhanced electric field treatment with Zn (LEEFT-Zn) near the anode. For experiments evaluating the electric field treatment alone, the zinc wire was replaced with a stainless-steel wire.

Two bacterial species, *S. epidermidis* and *Escherichia coli* (ATCC 10798), were selected as Gram-positive and Gram-negative models. Cell culture is provided in the Supporting Information. The obtained bacterial suspensions (∼10^8^–10^9^ CFU/mL) were washed three times with DI water and diluted 100-fold in water with different conductivities as influent or in Zn solution to achieve final influent concentrations of ∼10^6^–10^7^ CFU/mL. The influent conductivity was adjusted using sodium sulfate (Na_2_SO_4_), measured using an Orion Versa Star Pro conductivity probe (Thermo Scientific), and maintained between 1 and 200 μS/cm. The influent conductivity was maintained at ∼5 μS/cm using Na_2_SO_4_, sodium nitrate (NaNO_3_), sodium bicarbonate (NaHCO_3_), and sodium chloride (NaCl) to evaluate the effect of common anions on disinfection performance.

To evaluate the effect of Zn ions, a constant voltage was maintained at 1.5 V using a power source (Keithley 2400 SourceMeter). To evaluate the effect of electric field strength, a constant current of 0.9 mA was applied to maintain the Zn concentration at ∼3 mg/L. To evaluate the role of ROS, the ROS scavenger *t-*butanol (200 mM) was added into the influent.^33^ The flow rate was maintained at 5 mL/min using a peristaltic pump (MasterFlex L/S). To ensure maximum uptake of Zn by the bacteria and consistency of control and reactor experiments, the effluents were incubated for 2 h before plating. Bacterial concentrations in influent and effluent samples were quantified using standard plate counting. Zn concentrations in the effluent were measured using an atomic absorption spectrometer (PerkinElmer PinAAcle 900F, with PerkinElmer S10 Autosampler). Some samples were filtered through 0.22 μm syringe filters to remove the suspended bacterial cells so that the concentrations of the total Zn, the dissolved Zn in the solution, and the Zn adsorbed or taken by the cells can be determined.

### Electric Field Strength and Zn Concentration Simulation

The electric field strength and Zn concentration simulation in the bench-scale coaxial reactor was conducted using COMSOL Multiphysics. Laminar flow and electrostatics were solved to obtain the velocity field and electric potential. The electric field of the cross-section of the chamber is simulated by electrostatic module in a 3D model. Electric field (*E*) is defined by eq 1:

E=−∇V 1

where *V* is the electric potential.

Zn release from the anode was represented as a prescribed inward molar flux on the central electrode surface, computed from the measured current using Faraday’s law. Zn ions are transported by diffusion, electromigration, and convection. The inlet Zn concentration was set to zero, outlet used convective flux, solid walls were no-flux, and the anode applied the inward flux from Faraday’s law. The molar flux of Zn and boundary condition at the anode (Faraday flux) are calculated by eqs 2–5.

$$\nabla (-D_{\mathrm{Zn}}\nabla c_{\mathrm{Zn}}-z_{\mathrm{Zn}}\mu_{m}Fc_{\mathrm{Zn}}\nabla V)+\mu \nabla c_{\mathrm{Zn}}=R_{\mathrm{Zn}}$$ 2

$$N_{\mathrm{Zn}}=-D_{\mathrm{Zn}}\nabla c_{\mathrm{Zn}}-z_{\mathrm{Zn}}\mu_{m}Fc_{\mathrm{Zn}}\nabla V+\mu \nabla c_{\mathrm{Zn}}$$ 3

$$\mu_{m}=\frac{D_{\mathrm{Zn}}}{RT}$$ 4

$$-nN_{\mathrm{Zn}}=N_{0,\mathrm{anode}}$$ 5

where *D*_Zn_ denotes the diffusion coefficient, *c*_Zn_ is the molar concentration of Zn ions, *z*_Zn_ is the charge number of Zn ions (*z*_Zn_ = 2 for Zn^2+^), *u*_m_ is the ionic mobility (*u*_m_ = *D*_Zn_/*RT*), *F* is Faraday’s constant, *V* is the electric potential, μ is the fluid velocity vector, and *R*_Zn_ is the volumetric reaction term for the Zn species. The boundary flux condition uses −*n*·*N*_Zn_ = *N*_0,anode_, where *n* is the outward unit normal and $N_{0,\mathrm{anode}}=\frac{I}{z_{\mathrm{Zn}}FA}$ represents the prescribed inward molar flux of Zn ions at the surface of the central electrode.

### Flow Cytometry Analysis

The flow cytometry combined with two fluorescence staining dyes was employed to simultaneously enumerate total cell counts and evaluate the bacterial integrity in water samples.^34^ In each assay, 1 mL of sample was stained with 12 μL of fluorescent dye solution containing Invitrogen SYBR Green I (SG) (1:100 SG diluted in 10 mM Tris–HCl (pH 8.5, Bioworld) combined with Molecular Probe PI (3 μM final concentration).^35^ The samples were then incubated in the dark for 15 min and dispersed by oscillation for 30 s.^36^ All samples were processed in triplicate with one blank (i.e., unstained DNase/RNase-Free Distilled Water (Thermo Fisher Scientific)). Three positive controls (i.e., fresh pure culture of *S. epidermidis* or *E. coli*) and three negative controls (i.e., sodium hypochlorite-treated pure culture) were processed in parallel with the samples to separate the intact and damaged populations. The measurement was performed at medium flow rate (30 μL/min) for 1 min with a 50 mW solid-state laser emitting light at a fixed wavelength of 488 nm. Green and red fluorescence intensities were collected in the B525 channel (525 ± 40 nm) and B690 channel (690 ± 50 nm), respectively, along with sideward (SSC) and forward (FCS) scatter light intensities (Table S1). All flow cytometry standard (FCS) files were processed in FlowJo software (FlowJo LLC).

### Bacteria Inactivation Experiments Using a Scale-Up EFT-Zn Reactor

A scaled-up LEEFT-Zn reactor was fabricated with nine coaxial flow chambers connected electrically in parallel and hydraulically in series. The chambers were mounted between two custom fabricated PVC manifolds to ensure sequential flow across all nine chambers. Bacterial suspensions of *S. epidermidis* and *E. coli* were prepared as described previously and diluted 1000-fold in influent to get a final concentration of ∼10^5^–10^6^ CFU/mL. The influent conductivity was adjusted to 5, 50, and 100 μS/cm using Na_2_SO_4_. To maintain a stable effluent Zn concentration, constant current was applied and scaled proportionally with the flow rate. Specifically, currents of 30, 45, and 90 mA were applied at flow rates of 140, 210, and 420 mL/min, respectively, using a power source. Operational conditions and plating procedures were kept consistent with those used for the bench-scale reactor. For real water disinfection experiments, river and lake water samples were collected from South Fork Peachtree Creek and Candler Lake in Decatur, Georgia. The river water has a conductivity of 138.6 μS/cm and a pH of 7.2 and the lake water has a conductivity of 113.5 μS/cm and a pH of 7.4. The collected waters were treated directly using the scale-up system without any pretreatment. Bacterial concentrations in influent and effluent samples were quantified by culturing on LB agar plates at 37 °C for 24 h.

## Results and Discussion

### Operando Investigation of EFT-Zn Synergy Using the LOAC Device

The synergistic interaction between EFT and Zn was first tested using a LOAC device under operando conditions (Figure 1a). LOAC platform minimizes chemical and material usage and reduces time and spatial demands, which is suitable for operando mechanistic studies. In this work, a LOAC device with curved electrodes was designed to generate a linear electric field gradient along the *x*-axis (Figures 1b and S1). *S. epidermidis* cells were immobilized on the chip surface for proof-of-concept study. Zinc nitrate solutions of varying concentrations were introduced into the microchannel for Zn treatment, and the chip was connected to a pulse generator to apply controlled EFT conditions. Following treatment, samples were incubated for 2 h before staining with PI to assess membrane integrity. For imaging, the chip was mounted on a fluorescence microscope. A 440 μm × 20 μm region was cropped from each image for quantitative analysis (Figure 1b).

**Figure 1:**
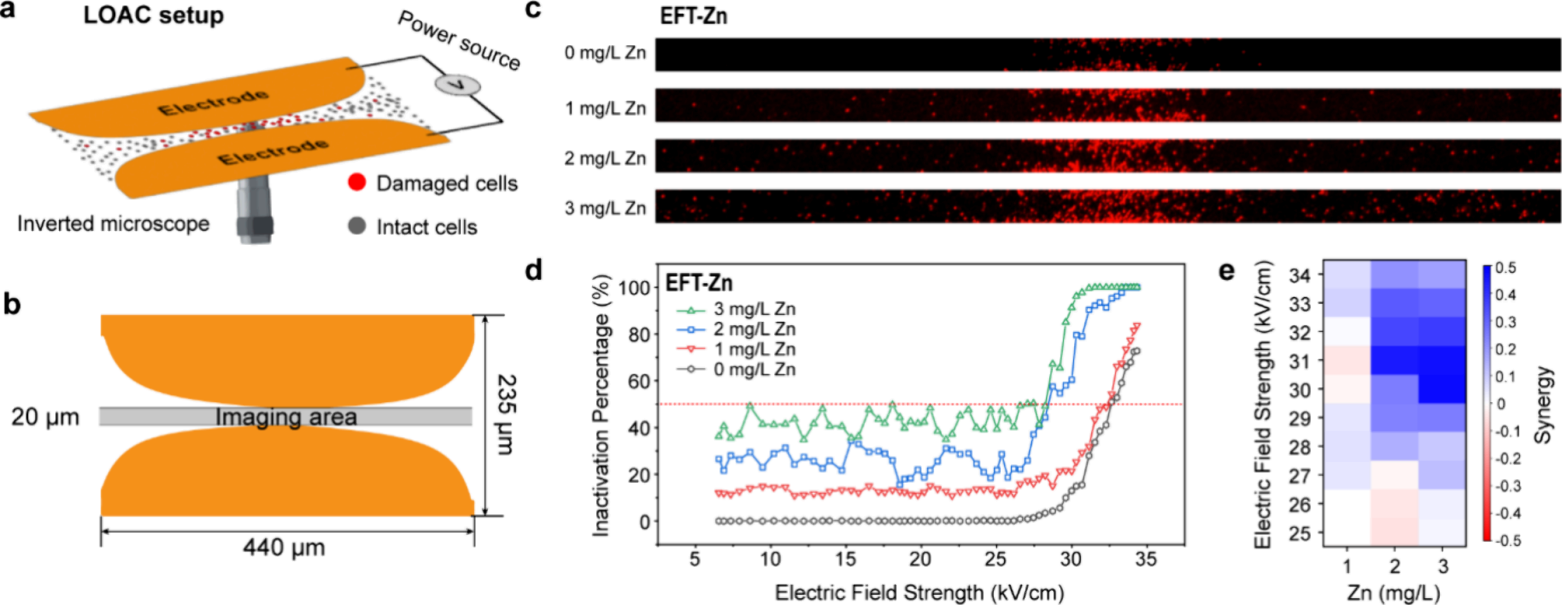
Operando investigation of the synergistic antibacterial effect between EFT and Zn using the LOAC device. (a) Schematic illustration of the LOAC experimental setup. (b) Geometry and dimensions of the chip. The shaded region (440 μm × 20 μm) indicates the area used for quantitative analysis. (c) Representative fluorescence images showing *S. epidermidis* inactivation under varying Zn concentrations and electric field strengths. Red fluorescence corresponds to PI-stained, inactivated cells. (d) Inactivation efficiencies of EFT-Zn treatment. The red dashed line indicates the lethal electroporation threshold where overall inactivation reaches 50%. (e) Synergy values (*S*) calculated through BIM for the combined EFT-Zn under different electric field strengths (25–34 kV/cm).

The inactivation performance of Zn alone was first tested on the LOAC device to assess the intrinsic antibacterial effect of Zn ions (Figure S2). It shows that 1 mg/L Zn results in ∼14% inactivation of *S. epidermidis*, which increases to ∼54% at 5 mg/L Zn. These results indicate that Zn exhibits moderate antibacterial activity under the tested conditions. When combined with EFT, a clear enhancement in bacterial inactivation is observed. Fluorescent images and the corresponding inactivation efficiencies under various EFT-Zn conditions are shown in Figure 1c,d, respectively. At low electric field strengths, the inactivation efficiency remains relatively unaffected. However, once the field intensity exceeds ∼25 kV/cm, a sharp increase in inactivation occurs. A complete inactivation (100%) is achieved at 31 kV/cm with 3 mg/L Zn, and ∼90% inactivation is observed with 2 mg/L Zn (Figures 1d and S3). In contrast, EFT alone could only achieve 28% inactivation at the same electric field strength. These results clearly demonstrate a strong synergistic effect between EFT and Zn treatment.

To quantify this synergy, the Bliss Independence Model (BIM) was applied. BIM model enables assessment of whether the combined effect of two treatments is synergistic, additive, or antagonistic.^37,38^ The BIM analysis was performed using eq 6, where all input values were expressed as the fraction of surviving bacteria.

$$S=\left(\frac{f_{\mathrm{EFT}}}{f_{\mathrm{Control}}}\right)\left(\frac{f_{\mathrm{Zn}}}{f_{\mathrm{Control}}}\right)-\frac{f_{\mathrm{EFT}-\mathrm{Zn}}}{f_{\mathrm{Control}}}$$ 6

where *f*_EFT_ refers to the survival fraction under EFT-only conditions, *f*_Zn_ refers to the survival fraction under Zn-only conditions, *f*_EFT‑Zn_ refers to the survival fraction under combined EFT-Zn treatment, and *f*_Control_ is the survival fraction untreated condition. The resulting synergy coefficient *S* quantifies the interaction type. *S* > 0 indicates a synergistic effect, *S* = 0 indicates an additive response, and *S* < 0 indicates antagonistic interaction.

As shown in Figure 1e, the *S* values for 1 mg/L Zn are close to zero across all electric field strengths, indicating an additive response. In contrast, positive *S* values are observed at 2 and 3 mg/L Zn, particularly at higher electric field strengths. The maximum synergy coefficients reach 0.444 for 2 mg/L Zn at 31 kV/cm and 0.472 for 3 mg/L Zn at 30 kV/cm, indicating strong synergistic interactions. At even higher electric field intensities, the *S* values slightly decrease, likely due to the near-complete inactivation, which limits the numerical accuracy of the BIM calculation. Overall, the distinctly positive *S* values confirm a robust synergy between EFT and Zn treatment and show high promise to achieve efficient bacterial inactivation within a safe Zn concentration range.

### Continuous-Flow Bench-Scale Water Disinfection Using EFT- Zn Treatment

After confirming the EFT-Zn synergy in microscale static conditions, we next evaluated its performance in a bench-scale continuous-flow system. The antimicrobial activity of Zn alone in solution was first assessed using two model bacterial strains, Gram-positive *S. epidermidis* and Gram-negative *E. coli*. In contrast to the results observed in the LOAC device, Zn exhibits markedly weaker antibacterial efficacy in the bulk liquid phase and there is no significant inactivation detected for both *S. epidermidis* and *E. coli* at Zn concentrations as high as 100 mg/L (Figure S4). Instead, a slight improvement in bacterial viability is observed, which aligns with previous studies reporting that moderate Zn concentrations (10 mM) can promote bacterial growth (*Bacillus cereus*) due to its nutritional property.^17^ At even higher Zn concentrations (>100 mg/L), inactivation become more evident, reaching ∼66% for *S. epidermidis* and ∼52% for *E. coli* at 10,000 mg/L. The discrepancy between LOAC and solution-based results may be attributed to the use of poly-l-lysine for cell immobilization on the chip, which can alter membrane properties through lipid immobilization and charge neutralization,^39,40^ potentially affecting bacterial susceptibility to external stimuli.

Next, the EFT-Zn performance was tested using a continuous-flow reactor (Figures 2a and S5) at an applied voltage of 1.5 V. The reactor consists of a 21.5 cm-long stainless steel cylindrical cathode with a 5 mm inner diameter and a Zn wire with a diameter of ∼355 μm as the central anode. Electric field simulation shows a gradient of the electric field strength inside the reactor with a highly enhanced electric field strength (∼21 kV/m) at central anode surface (Figure 2b). This configuration enables a LEEFT-Zn near the anode. The hydraulic retention time (HRT) was maintained at 1 min, which corresponds to a flow rate of 5 mL/min, to ensure bacterial cells transported into this high electric field region.^32^ The solution conductivity was adjusted to achieve different Zn concentrations (Figure S6). As shown in Figure 2c,d, the inactivation efficiency for both bacterial species increases with Zn concentrations. Under pure LEEFT conditions (Zn = 0 mg/L, obtained using a stainless steel wire anode), only ∼25% inactivation is achieved. However, the inactivation efficiency increases significantly for Zn higher than 1 mg/L, reaching ∼2.5 log (∼99.7%) inactivation for both bacterial species at 3 mg/L Zn, which is significantly higher than that obtained from Zn-only treatment or sequential LEEFT and Zn treatments with same Zn concentrations (Figure S7). These results confirm a strong synergistic effect between the applied electric field and Zn under continuous-flow conditions. However, it should be noted that the highest simulated electric field strength in this bench-scale reactor (∼21 kV/m, Figure 2b) is significantly lower than the field intensity required for synergy in the LOAC device (∼30 kV/cm). The potential reasons for this discrepancy are discussed further in following section.

**Figure 2:**
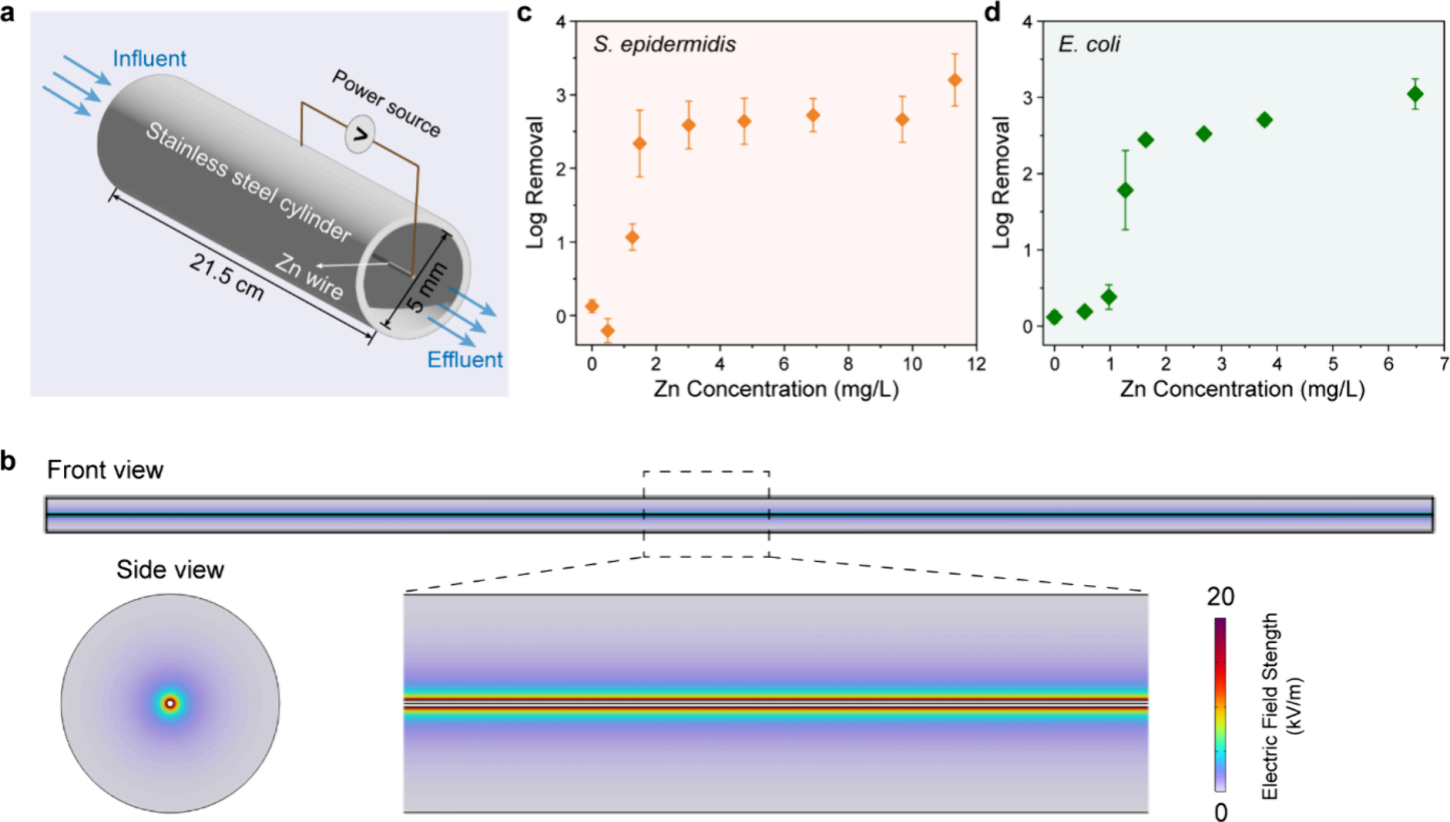
The bench-scale LEEFT-Zn reactor design and inactivation performance. (a) Schematic illustration of bench-scale LEEFT-Zn reactor setup. (b) Electric field distribution within the rector simulated using COMSOL Multiphysics. (c) Inactivation efficiency of *S. epidermidis* under LEEFT-Zn treatment at a fixed electric field strength with different Zn concentrations. (d) Inactivation efficiency of *E. coli* under LEEFT-Zn treatment at a fixed electric field strength with different Zn concentrations.

To investigate the effect of electric field strength, effluent Zn concentrations were maintained at ∼3 mg/L by applying a constant current of 0.9 mA (Figure S8). Water conductivity was then adjusted to generate different system voltages (Figure S9). As shown in Figure 3a,b, the inactivation efficiencies for both *S. epidermidis* and *E. coli* increase with increasing voltage under fixed Zn conditions. At a conductivity of 5 μS/cm, EFT-Zn shows similar inactivation efficiencies across different common anion conditions (Figure S10), and these ions do not significantly affect Zn ion release in the system (Figure S11). The inactivation performance was further evaluated by spiking both bacterial strains into two real waters. The observed inactivation efficiencies are ∼2.3 log (Figure S12), which are comparable to those obtained in synthetic water matrices. However, it should be noted that, even at extremely low voltage (∼0.1 V), the system achieves 1.36 log removal for *S. epidermidis* and 1.91 log removal for *E. coli*, still significantly higher than that observed for Zn-only treatment. As previously mentioned, the electric field strengths corresponding to these low-voltage conditions under continuous-flow conditions are far below the critical threshold determined using the LOAC device. On the LOAC platform, Zn is uniformly distributed at low bulk concentrations (<5 mg/L), and cell damage was quantified using PI staining following a 2 h post-treatment incubation to ensure only dead cells were counted. Therefore, the critical electric field strength (∼30 kV/cm) identified on the LOAC device represents electroporation, where transient or permanent membrane pores are formed. This threshold remains nearly constant across Zn concentrations below 5 mg/L (Figure 1d), indicating that microbial inactivation in the LOAC system requires strong electric fields to facilitate sufficient intracellular Zn accumulation. In contrast, the bench-scale device operates in a highly nonuniform Zn condition. The local Zn concentrations near the central electrode are significantly higher than the bulk concentrations, reaching around 10,000 mg/L when the bulk concentration is fixed at 3 mg/L (Figures 3c and S13). Under these conditions, synergistic effects may not rely solely on electroporation to overcome Zn transport limitations. Instead, even moderate electric fields can induce sublethal membrane perturbations that increase permeability. Previous studies have shown that bacterial lipid membranes are dynamic structures that can exhibit functional and structural disruption at field strengths as low as tens of kV/m range.^23,41^ At elevated local Zn concentrations, such sublethal membrane disruptions likely enhance Zn ion penetration and intracellular accumulation, improving antibacterial efficiency even under relatively weak electric field conditions. Correspondingly, the fraction of dissolved Zn decreased from 98.8 and 97.9% under Zn-only conditions to 78.4 and 72.3% under EFT-Zn conditions for *S. epidermidis* and *E. coli*, respectively, indicating enhanced Zn association with bacterial cells in the presence of an electric field (Figure S14). Moreover, in the LOAC experiments, poly-l-lysine was used for cell immobilization, which may affect membrane response to external stimuli. As such, the absolute critical electric field threshold identified in the LOAC platform may be specific to the immobilized state and contribute to the differences observed between the LOAC and continuous-flow system.

**Figure 3:**
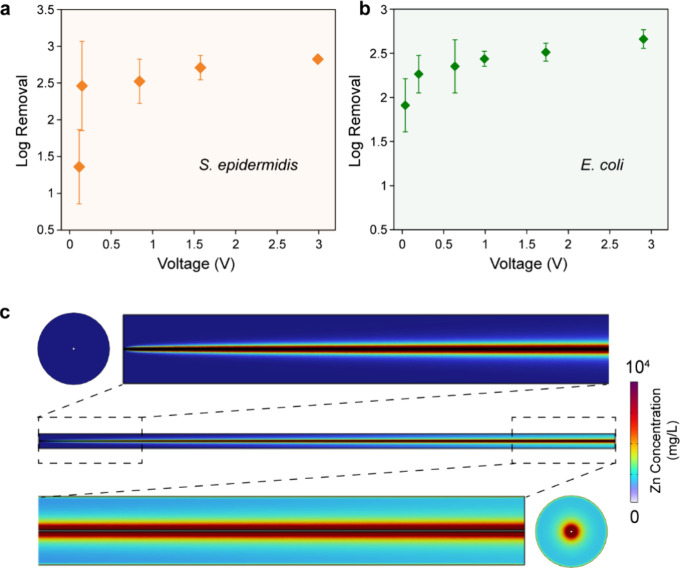
Disinfection performance of the bench-scale LEEFT-Zn reactor under controlled Zn conditions. Inactivation efficiency of (a) *S. epidermidis* and (b) *E. coli* at a fixed effluent Zn concentration (∼3 mg/L) under varying operating voltages. (c) Simulated Zn concentration within the LEEFT-Zn reactor after stabilization, obtained using COMSOL Multiphysics.

To further confirm that bacterial inactivation was primarily achieved through EFT-Zn synergy rather than electrochemical oxidation, a ROS scavenger, *t-*butanol, was added to the influent. The addition of *t-*butanol does not lead to a noticeable decrease in removal efficiency (Figure S15), suggesting that ROS generation is not the dominant inactivation mechanism. Flow cytometry analysis was further conducted to assess bacterial membrane integrity following treatment. The results reveal that in the effluent, 99.7% of *S. epidermidis* and 93.9% of *E. coli* cells exhibit membrane damage (Figure S16). Importantly, the total cell counts in the effluents only have slight decrease, suggesting that the cells are not lysed or lost during treatment. These findings, along with the plate counting results, collectively demonstrate that bacterial inactivation in the EFT-Zn system primarily arises from the physical synergistic effect between electric field and Zn, rather than from electrochemical oxidation, ROS-mediated processes, or sorption to the electrodes.

### Scale-Up of the EFT-Zn System for High-Throughput Water Disinfection

While the bench-scale reactor effectively demonstrated EFT-Zn disinfection performance, its throughput (5 mL/min) is insufficient for practical applications. To enable system scale-up without compromising electric field strength, a modular multichamber LEEFT-Zn system was developed (Figure 4a–c). The system contains nine coaxial flow chambers arranged in parallel between two end holders. Each chamber is 50 cm in length with an inner diameter of 10 mm. The overall device dimension is approximately 60 cm × 15 cm × 15 cm, which allows the system to be placed on a portable cart for point-of-care deployment (Figure S17). All nine stainless steel cylindrical chambers were electrically connected using conductive tape to serve as cathodes, while the central Zn wires were connected as anodes. A voltage or current was applied to generate parallel electrical circuits across all chambers (Figure S18), ensuring uniform electric field strength and Zn release rates among them. The hydraulic configuration was designed such that water sequentially flowed through each chamber in series (Figure 4b), effectively extending the treatment path. This configuration enables a significant increase in total flow capacity without sacrificing disinfection performance or energy efficiency.

**Figure 4:**
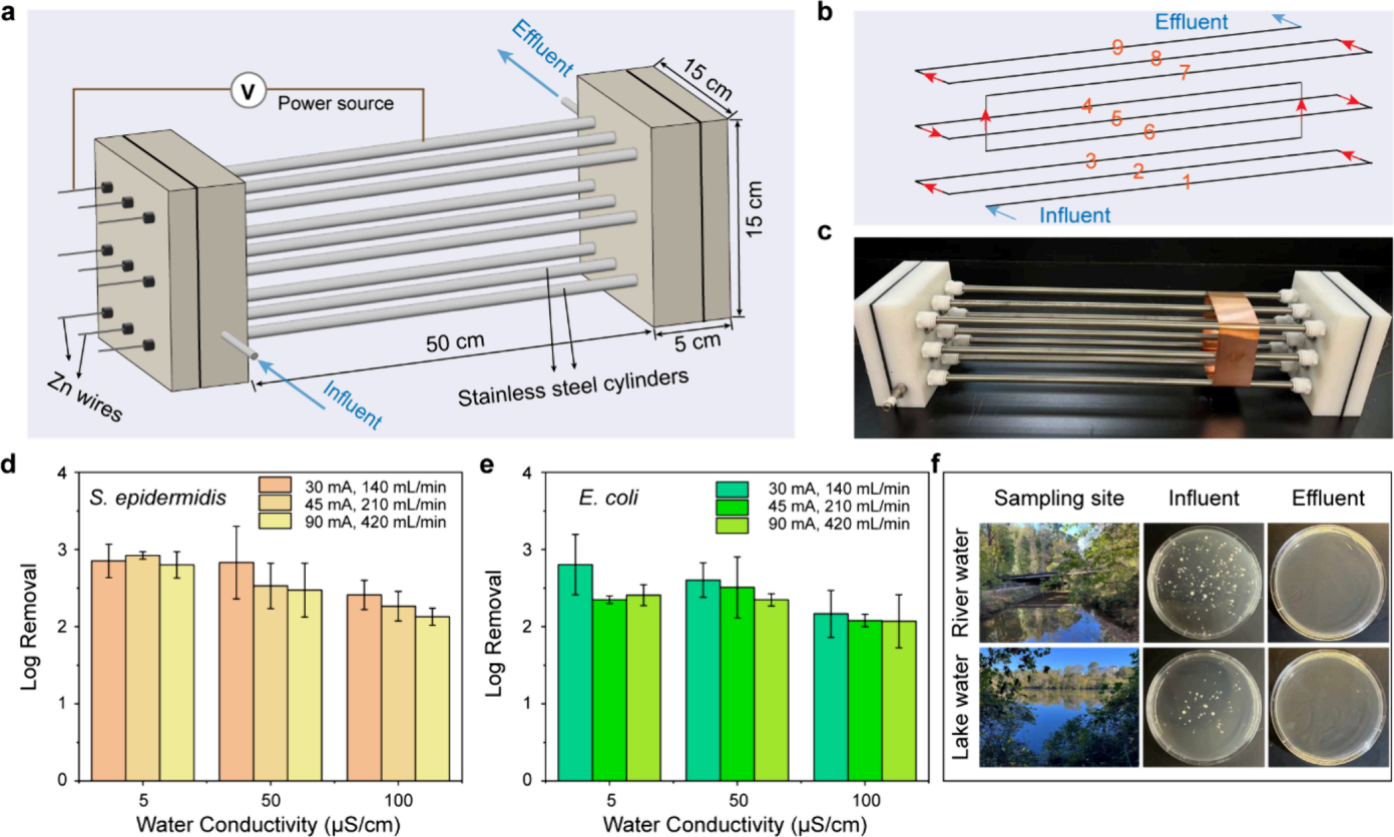
Scaled-up LEEFT-Zn system and its disinfection performance. (a) Schematic illustration of the scaled-up LEEFT-Zn device composed of nine coaxial flow chambers arranged in parallel. (b) Serial water flows through each chamber. (c) Photograph of the fabricated LEEFT-Zn device. (d) Inactivation efficiency of *S. epidermidis* under various flow rates, currents, and water conductivities. (e) Inactivation efficiency of *E. coli* under these conditions. (f) Photographs of the river and lake water results, including sampling sites and incubations of untreated influent and LEEFT-Zn treated effluent samples.

Because Zn concentration is a key water quality concern for drinking water applications, constant current was applied to maintain a stable Zn level during operation. We first evaluated Zn release under different applied currents using water with conductivity of 5 μS/cm at a flow rate of 140 mL/min, corresponding to an HRT of 1.5 min. The results show that Zn is well correlated with the applied current and reaches a concentration of 3.19 mg/L at 30 mA (Figure S19a). Under this condition, the working voltage is 3.68 V (Figure S19b), and the system achieves 2.85 log and 2.8 log reductions for *S. epidermidis* and *E. coli*, respectively (Figure 4d,e), which are comparable to the performance observed in the bench-scale rector at similar Zn concentrations. To further enhance throughput, the flow rate was raised, and the applied current was proportionally scaled to maintain a stable Zn concentration. Specifically, flow rates of 210 and 420 mL/min, which correspond to HRT of 1 and 0.5 min, were tested with the applied currents of 45 and 90 mA, separately. Zn concentration remains stable under these conditions (Figure S20). However, the increased current leads to higher operating voltages, rising from 3.68 to 12.35 V (Figure S21). Despite the shorter treatment time at higher flow rates, the enhanced electric field intensity likely compensates for the reduced HRT, resulting in comparable inactivation efficiencies for both bacterial species across different flow conditions (Figure 4d,e).

It is important to note that real drinking water typically has a conductivity of ∼50–100 μS/cm. Our bench-scale experiments reveal that variations in conductivity can affect the operating voltage and treatment efficiency. To verify the applicability of the scaled-up LEEFT-Zn system for realistic water matrices, we further evaluated its performance using waters with conductivities of 50 and 100 μS/cm. Three flow rates (140, 210, and 420 mL/min) were tested, with corresponding currents of 30, 45, and 90 mA to maintain the consistent Zn levels (Figure S20). As expected, the system voltage decreases significantly with increasing conductivity, from 12.35 to 1.33 V at 420 mL/min when conductivity increases from 5 to 100 μS/cm. Despite this reduction in voltage, only a minor decrease in bacterial inactivation is observed, from 2.80 log to 2.12 log for *S. epidermidis* and from 2.41 log to 2.07 log for *E. coli* (Figure 4d,e). As discussed earlier, the extremely high localized Zn concentration near the anode likely facilitates cell membrane disruption even under very low electric field strengths. Finally, the system was tested using two untreated surface water samples under a flow rate of 420 mL/min and an applied current of 90 mA. A complete removal of viable bacteria was achieved in both natural waters (Figure 4f), demonstrating effective disinfection in complex microbial communities. Under this condition, the estimated energy consumption is approximately 17.1 J/L (Table S2), which is comparable to or lower than that of other water disinfection processes such as UV irradiation and ozonation.^42^ These results demonstrate that the scaled-up LEEFT-Zn system maintains robust disinfection performance under realistic water conditions, demonstrating its strong potential for practical water treatment applications.

### Environmental Implications and Perspectives

This study introduces a combined EFT-Zn process as an efficient, scalable, and mineral supplement strategy for drinking water disinfection. By coupling a mild electric field with trace levels of Zn, a strong synergistic effect between electric field and Zn was achieved, promoting bacterial inactivation while maintaining Zn concentrations within the range beneficial for human health. This synergistic effect was directly visualized and quantified using a LOAC platform and subsequently validated in a bench-scale continuous-flow reactor. A scaled-up EFT-Zn system was further developed to accommodate high-throughput operation and successfully demonstrated robust disinfection performance in natural surface waters.

To the best of our knowledge, this is the first study that harnesses the antimicrobial properties of Zn for drinking water disinfection applications. Although a wide range of antimicrobial metals have been identified, only a few, most notably Cu and Ag, have been applied for water treatment.^43–47^ However, their relatively high toxicity at effective disinfection doses has limited their practical use in potable water applications. The integration of an electric field enables the use of milder, more biocompatible metals, such as Zn, enhancing their antimicrobial properties without exceeding safe concentration thresholds. In contrast to Cu and Ag, Zn has a much higher permissible concentration in drinking water. The regulatory guidelines for Zn are primarily based on their aesthetic considerations rather than toxicity concern, which allows for easier operational control. Moreover, residual Zn in the treated water provides added nutritional value, serving as a beneficial source of essential micronutrients.

While the disinfection performance of the EFT-Zn system (∼3-log reduction) is lower than that of some reported photothermal or electrochemical disinfection techniques,^48–57^ those approaches typically rely on the generation or enhancement of ROS for microbial inactivation. Such ROS-driven processes have the potential to oxidize the chloride ions in water to form active chlorine species, ultimately leading to the formation of toxic chlorinated disinfection byproducts.^58^ In contrast, the EFT-Zn process operates primarily through a physical mechanism, minimizing reliance on chemical oxidations and potentially avoiding secondary contamination.

Nevertheless, further optimization is needed to fully realize the potential of the EFT-Zn under diverse real-world conditions. First, water chemistry parameters, such as pH, should be evaluated to define the operational range, as they may affect Zn speciation, release behavior, and disinfection performance. Second, while electrochemical oxidation has proven to be minimal in our system, a comprehensive assessment of the formation of disinfection byproducts, particularly in waters with different natural organic matters and chloride levels, is necessary to confirm the absence of secondary contamination. Third, the long-term operational stability of the Zn anode under continuous-flow conditions should also be tested. One potential strategy to extend electrode lifespan is to employ a nonconsumable substrate (e.g., stainless steel) with in situ electrodeposition of Zn to enable regeneration of the active electrode surface.^59^ Despite these considerations, the current system demonstrates stable and effective disinfection performance in natural surface waters, indicating its suitability for point-of-care applications or as the last step for drinking water treatment. Collectively, this study establishes a foundation for next-generation water treatment technologies that integrate microbial safety, sustainability, and human health enhancement through micronutrient supplementation.

## Supplementary Material



## Notes

Additional information on simulated electric field strength, inactivation efficiency of Zn ions and EFT-Zn in the LOAC device, inactivation efficiency of Zn ions in solution, photograph of the continuous-flow EFT-Zn reactors, inactivation efficiency of EFT-Zn in the reactors, simulated Zn concentrations, and flow cytometry analysis (PDF)
