# Supplementary material for: Drinking Water Disinfection Using Nutritional Level Zinc Assisted with Electric Field Treatment
Source: Environ Sci Technol. 2026 Apr 29;60(18):13734–43. doi: 10.1021/acs.est.6c03361 (PMC13173648; doi:10.1021/acs.est.6c03361)
Supplement: Supplementary file 1 [file es6c03361_si_001.pdf]

Supporting Information for

Drinking water disinfection using nutritional level zinc assisted with electric field treatment

Wei Wang, Mourin Jarin, Farshid Khan, Feiyang Mo, Shuai Wang, Kaiqin Bian, Anuja Tripathi, Ameet J Pinto, and *Xing Xie* *

School of Civil and Environmental Engineering, Georgia Institute of Technology, Atlanta, Georgia 30332, United States

*Corresponding Author: Xing Xie; Email: xing.xie@ce.gatech.edu

The Supporting Information contains 15 pages, 3 notes, 21 figures, and 2 tables.

Supplementary Note 1. Bacterial immobilization on the chip.

The chip was first coated with Poly-L-lysine for bacteria immobilization. Briefly, Poly-L-lysine was combined 1:1 with a 2 M pre-prepared borate buffer (composed 133 of 3.1 g Boric acid (Millipore, cat# 100765) and 0.5 g NaOH (Millipore, cat# SX0593-1)). An aliquot of 50 μL of the mixture was dropped onto the chip surface and culture for 2 h in a closed petri dish to avoid droplet evaporation. The remaining waters and chemicals were rinsed off gently with DI water, and then the chip was dried at 60 °C for 30 min and stored at 4 °C for further use. For bacterial immobilization, an aliquot of 50 μL of the harvest bacterial suspension was dropped on a Poly-L-lysine coated chip and incubated for 30 min. The unbounded cells were rinsed off gently with DI water.

Supplementary Note 2. Fluorescent image processing.

A 440 μm × 20 μm region was cropped from each image for quantitative analysis in MATLAB. The area was then vertically broken up into 120 columns, and the cell number in each column was counted using binary colour coding in MATLAB. The inactivation efficiency in each column was then calculated by dividing the total number of PI-labelled cells by the total number of cells present from the DIC image in every column. Up to 30 replicated channels were used for a certain calculation. As the channel is symmetrical, all quantified values have a duplicate. This analysis results in 60 values for each electric field strength data point shown in the results. The bulk of the results in the text reflect single data points that were calculated as the average of all 60 values gathered from each of 30 replicate channels.

Supplementary Note 3. Bacterial cultivation.

S. epidermidis and *E. coli* were cultured in nutrient broth (Becton Dickinson, cat# 234000) and Luria-Bertani (LB) broth (Miller, cat# 97064) separately. Bacterial cells were streaked onto the NB-agar or LB- agar plates and incubated at 37 °C for 24 h. A single colony was then transferred to 15 mL of the autoclaved NB or LB medium and incubated at 37 °C with agitation (200 rpm) for 14-16 h to get the bacterial suspension.

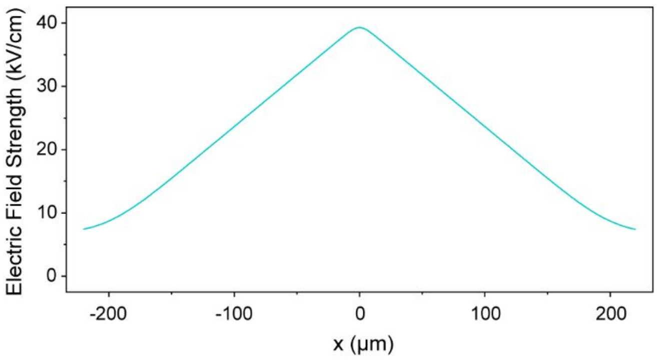

Figure S1. Simulated electric field strength distribution along the *x*-axis of the LOAC device.

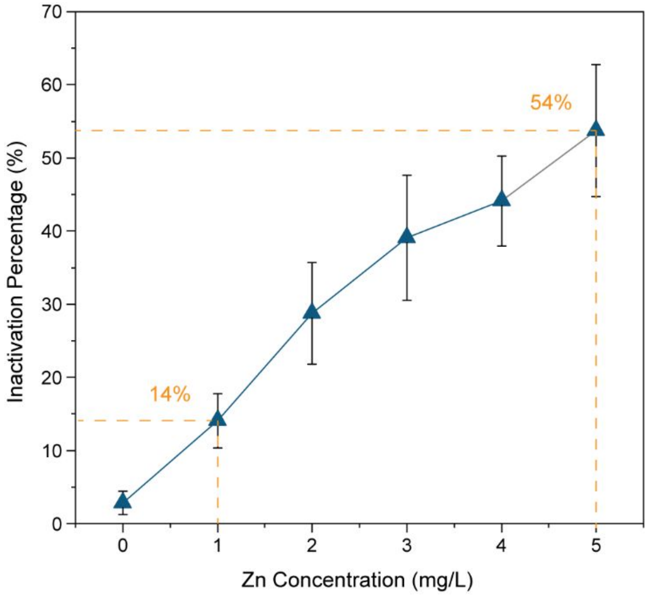

Figure S2. Inactivation efficiency of Zn ions applied individually in the LOAC device. Error bars represent 95% confidence intervals of the mean values from up to 30 replicate channels.

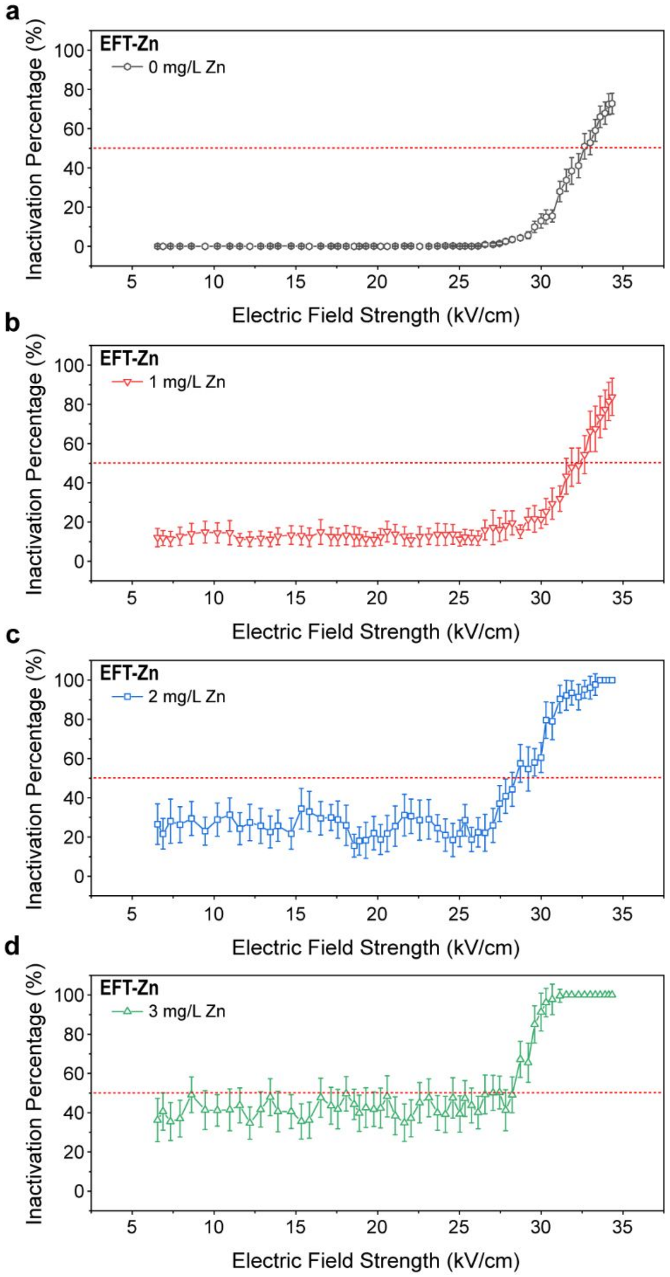

Figure S3. Inactivation percentages under combined EFT-Zn treatment with Zn concentrations of 0-3 mg/L. Error bars represent 95% confidence intervals from up to 30 replicate channels per condition. **(a)** 0 mg/L, **(b)** 1 mg/L, **(c)** 2 mg/L, and **(d)** 3 mg/L. The red dashed line indicates the lethal electroporation threshold where 50% inactivation is achieved.

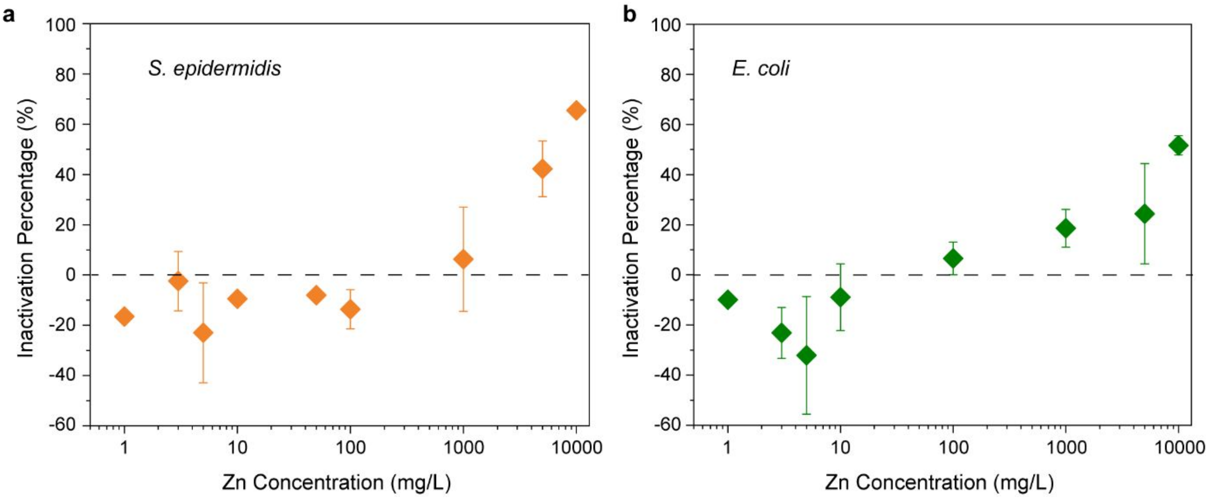

Figure S4. Bacterial inactivation efficiency under Zn only treatment in solution. (a) *S. epidermidis*, **(b)** *E. coli*. The dashed line indicates the influent concentration (0% removal).

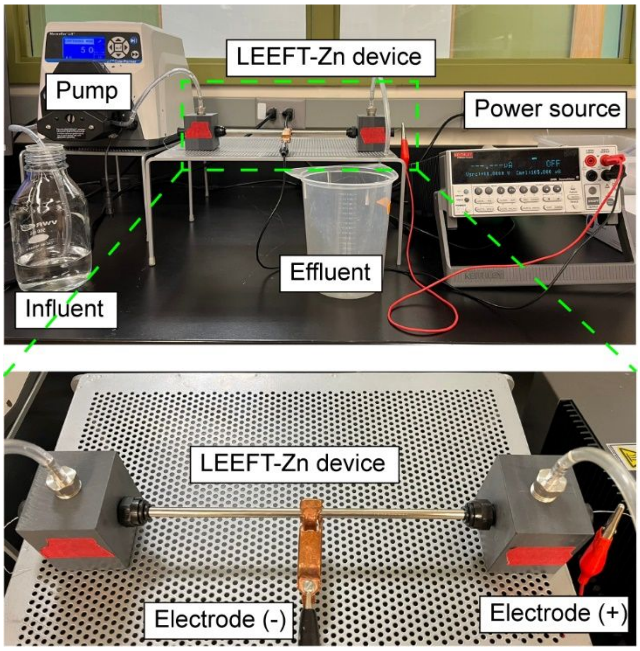

Figure S5. Photograph of the bench-scale continuous-flow EFT-Zn reactor.

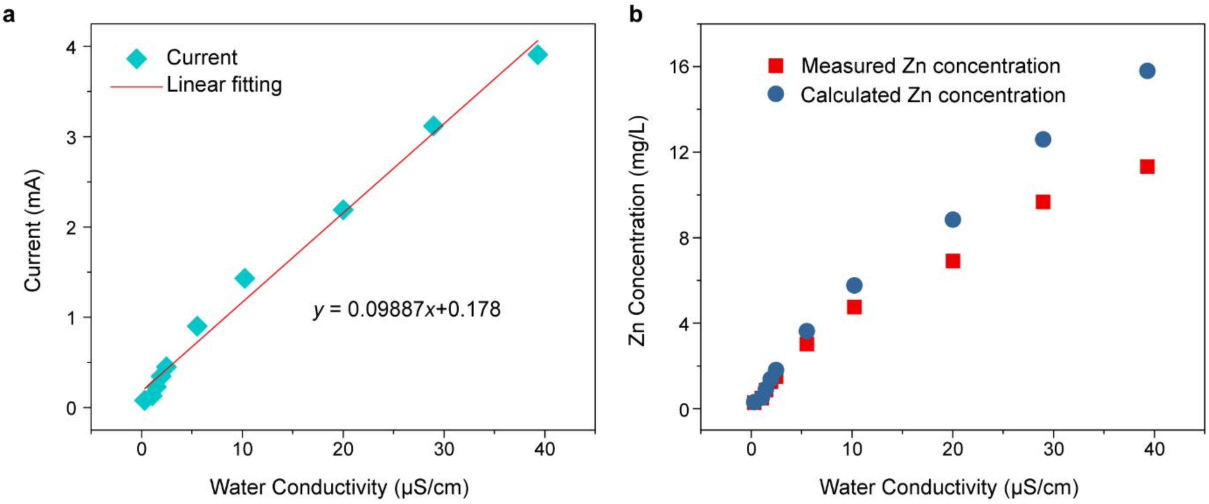

Figure S6. Effect of water conductivity on system current and effluent Zn concentration at an applied voltage of 1.5 V. (a) Correlation between water conductivity and the system current, **(b)** Measured Zn concentration under different water conductivities and the theoretical Zn concentration calculated from the Faraday's laws of electrolysis and the system current.

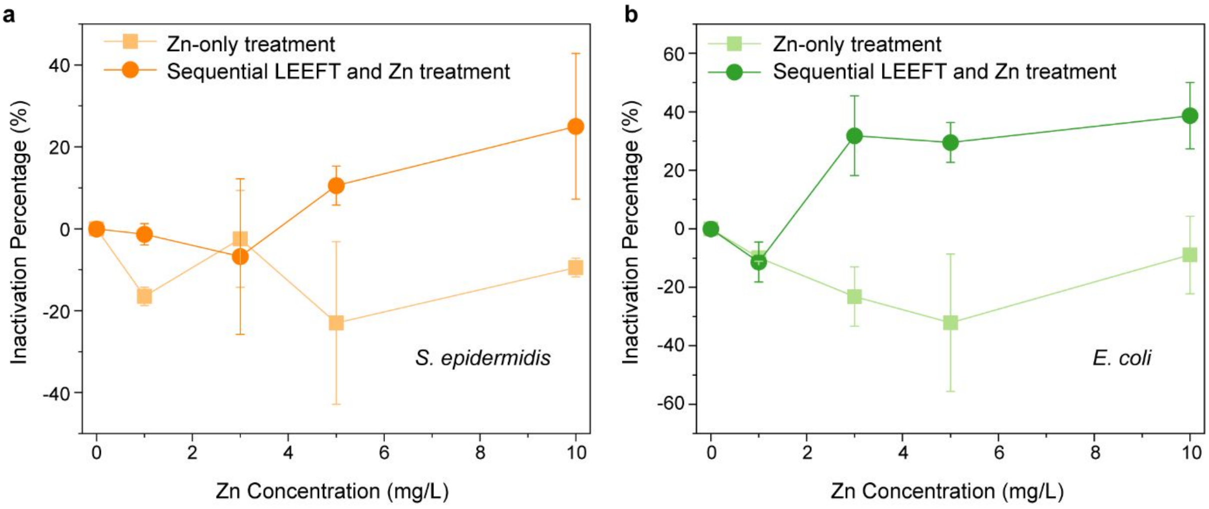

Figure S7. Bacterial inactivation efficiency under Zn-only treatment and sequential LEEFT and Zn treatment. (a) *S. epidermidis*, **(b)** *E. coli*.

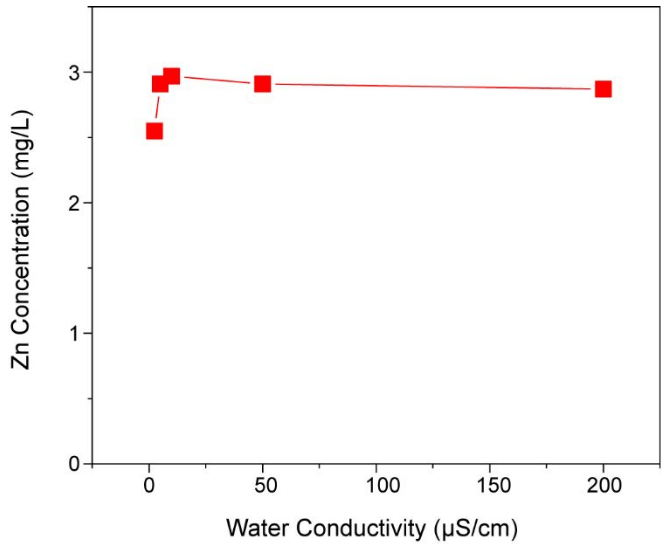

Figure S8. Effect of water conductivity on effluent Zn concentration at an applied current of 0.9 mA.

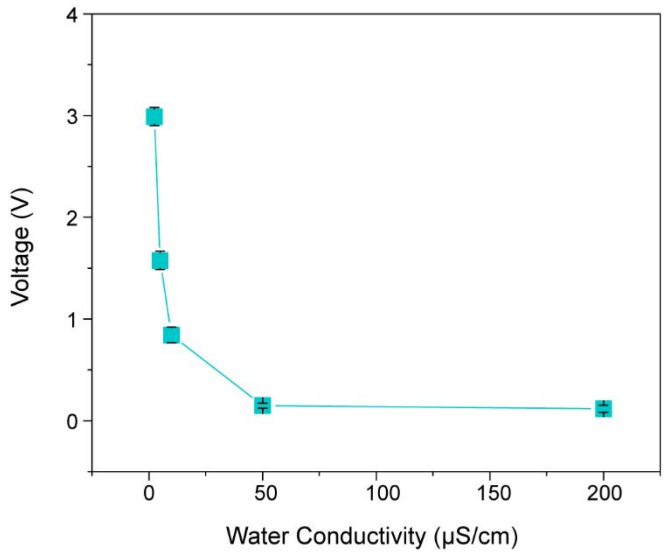

Figure S9. Effect of water conductivity on system voltage at an applied current of 0.9 mA.

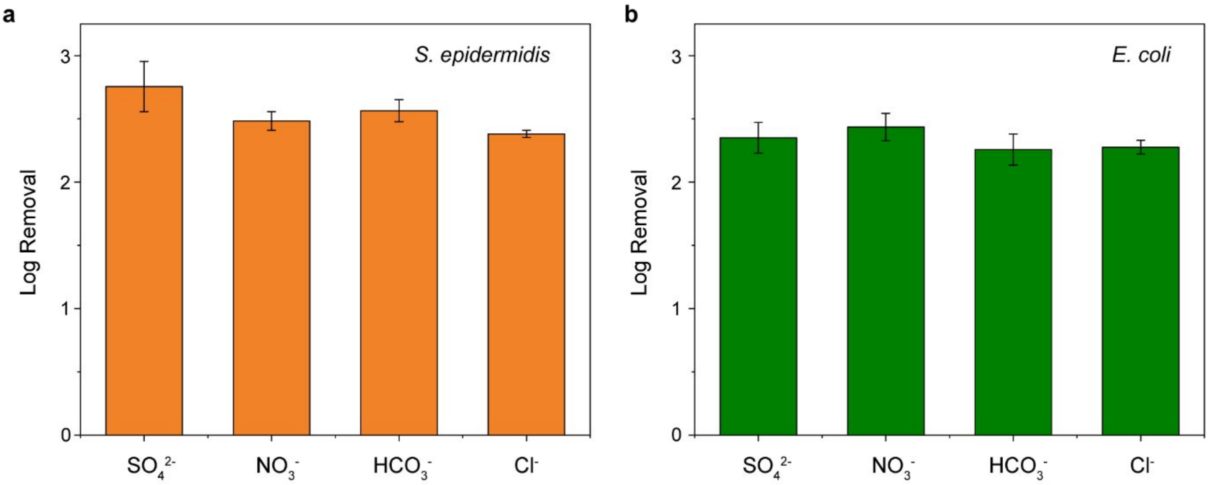

Figure S10. Effect of common anions on bacterial inactivation efficiency under EFT-Zn conditions at a conductivity of ~5 μS/cm. (a) *S. epidermidis*, **(b)** *E. coli*.

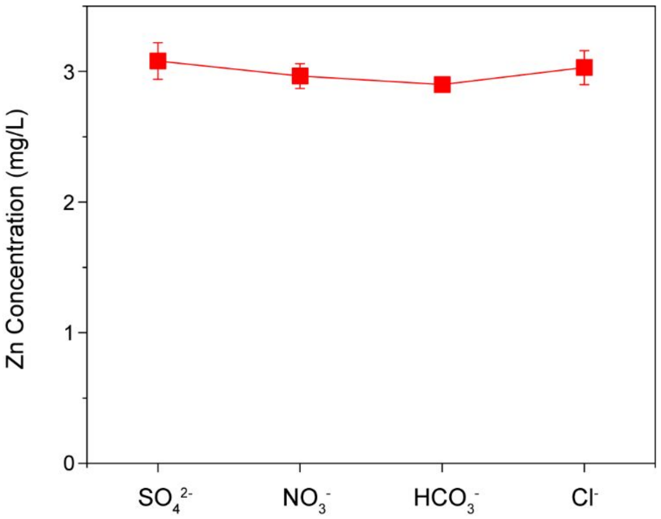

Figure S11. Effect of common anions on effluent Zn concentration under EFT-Zn conditions at a conductivity of ~5 μS/cm.

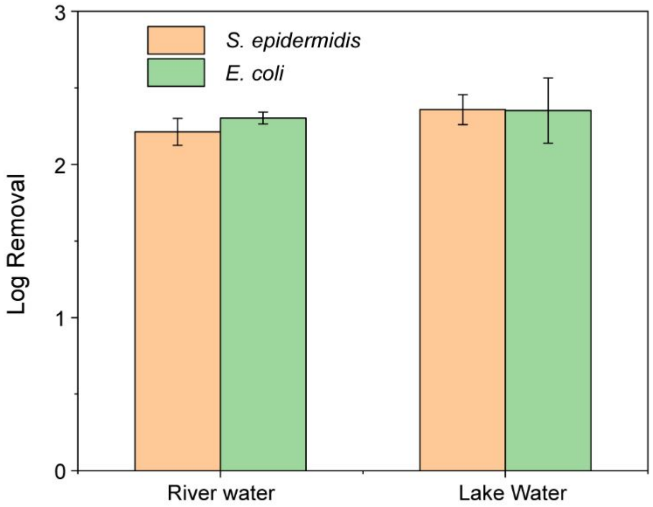

Figure S12. Inactivation efficiency of *S. epidermidis* and *E. coli* spiked into real water samples.

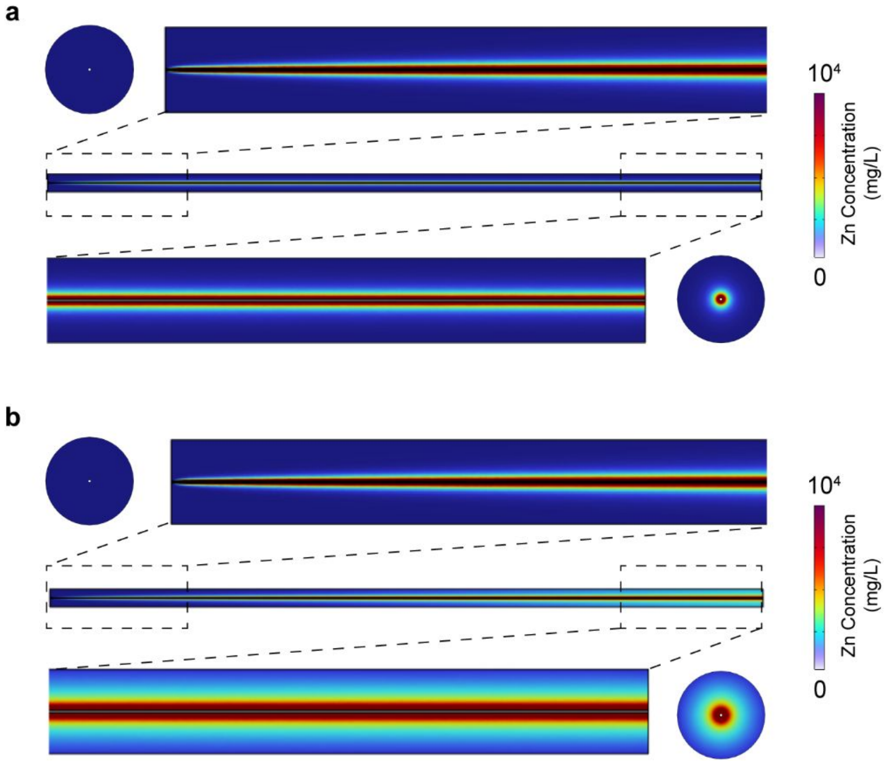

Figure S13. Simulated Zn concentration profiles within the LEEFT-Zn reactor using COMSOL Multiphysics at (a) 6 s and **(b)** 30 s of operation.

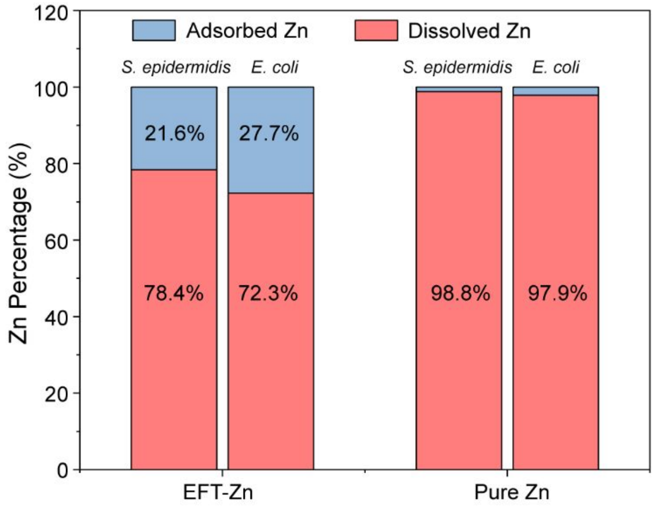

Figure S14. Distribution of dissolved Zn and absorbed Zn for *S. epidermidis* and *E. coli* under EFT-Zn and pure Zn treatment at a bulk Zn concentration of 3 mg/L.

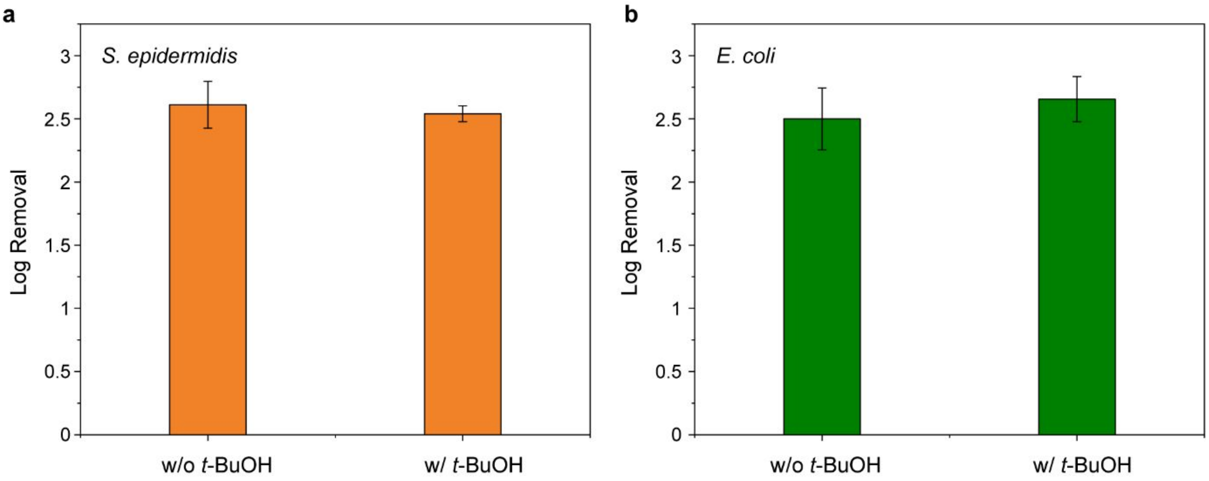

Figure S15. Comparison of inactivation efficiency in the presence and absence of ROS scavenger *n*-Butanol (200 mM). (a) *S. epidermidis*, **(b)** *E. coli*.

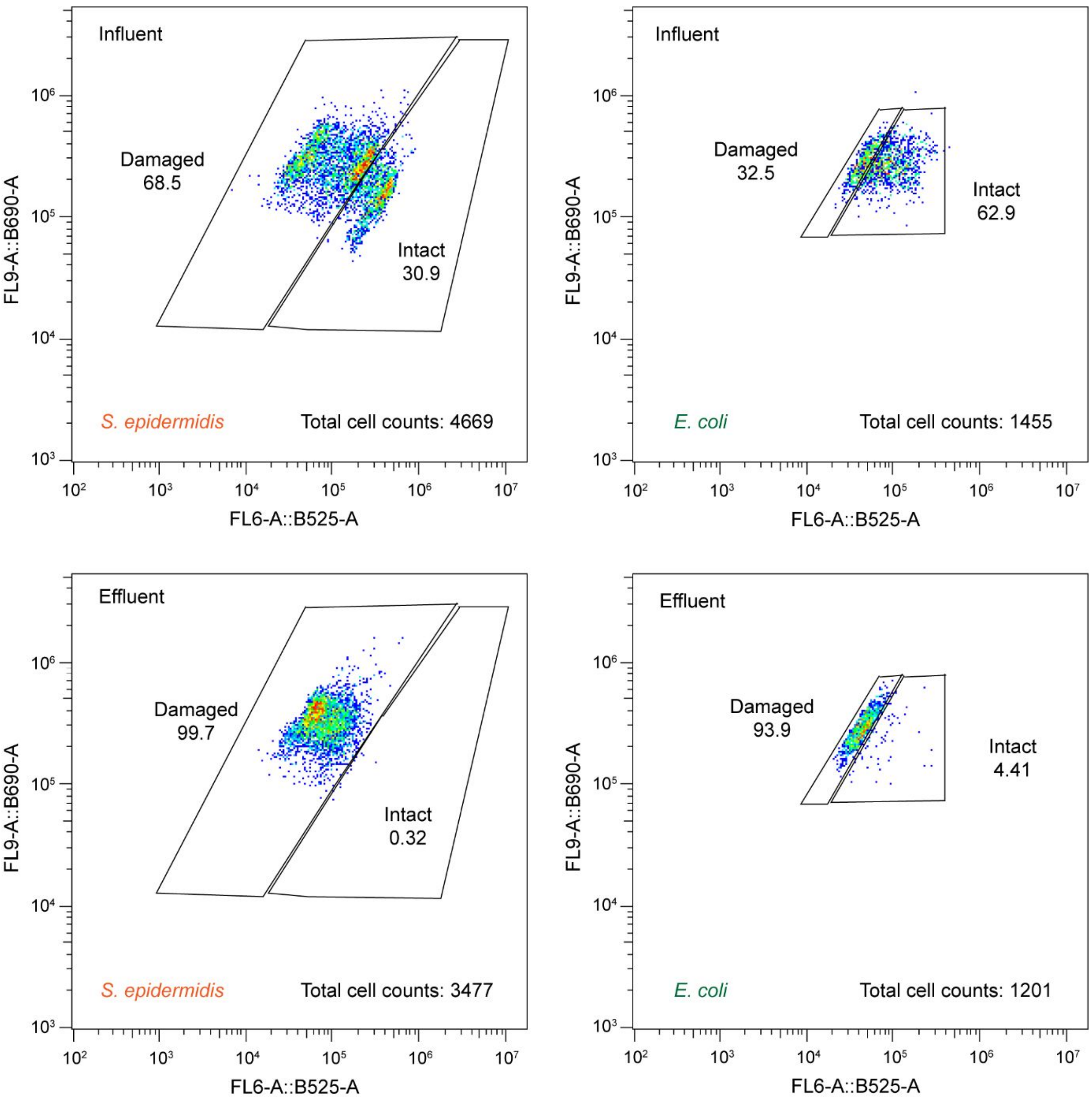

Figure S16. Flow cytometry analysis of bacterial cells in influent and effluent samples.

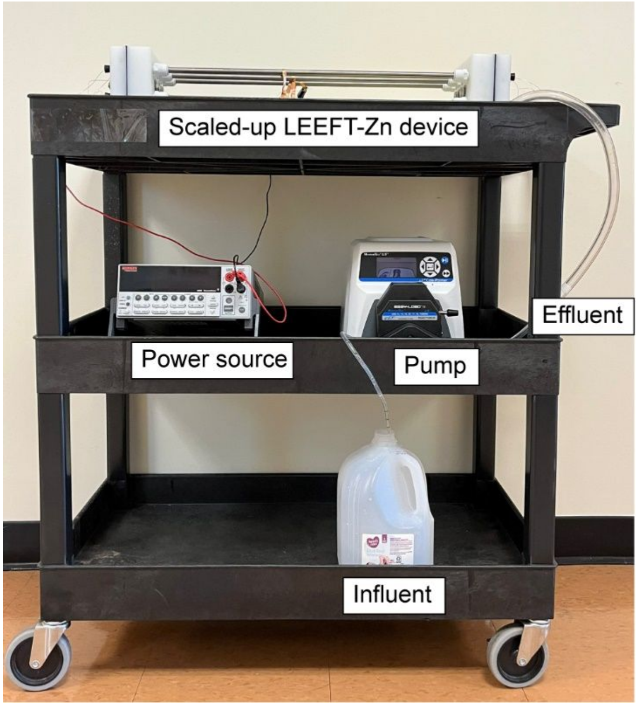

Figure S17. Photograph of the scaled-up EFT-Zn reactor on a portable cart.

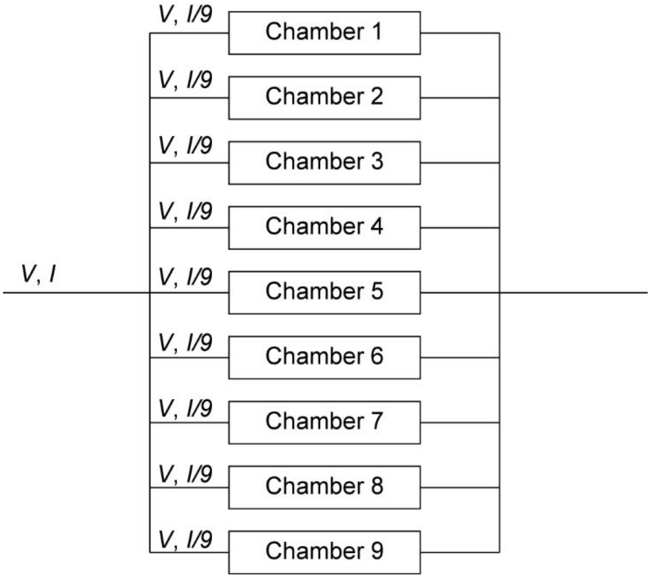

Figure S18. Parallel electrical circuit configuration of the nine-chamber scaled-up device. Each reactor has the same with the overall system voltage with current distributed equally (one-ninth of total system current).

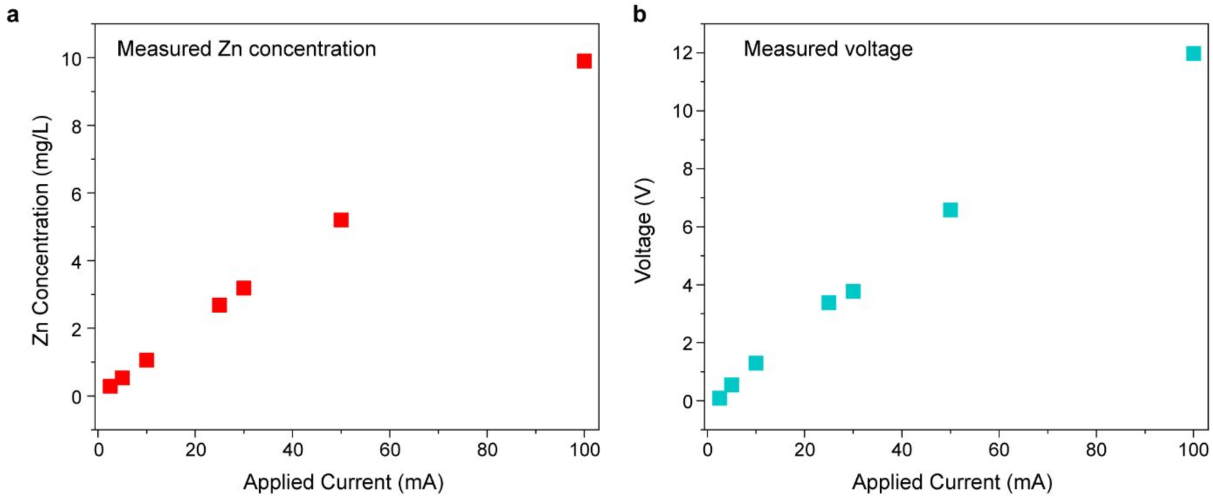

Figure S19. Effluent Zn concentrations and system voltages measured under different applied currents using the scaled-up reactor with 5 μS/cm water. (a) Zn concentration, **(b)** System voltage.

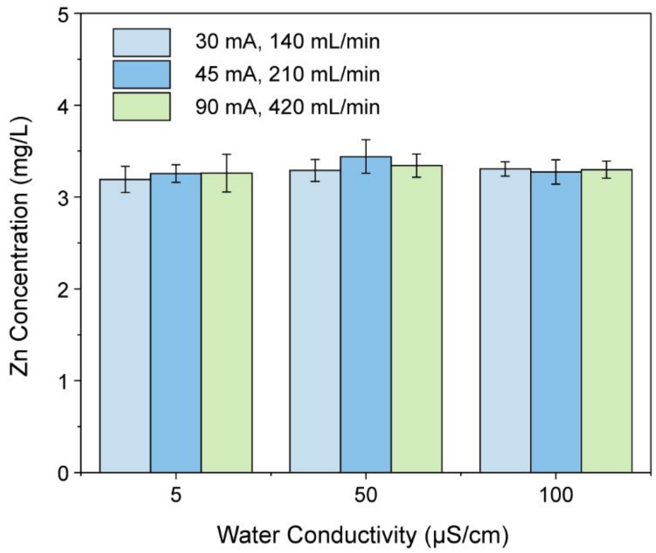

Figure S20. Effluent Zn concentrations under various flow rates, currents, and water conductivities.

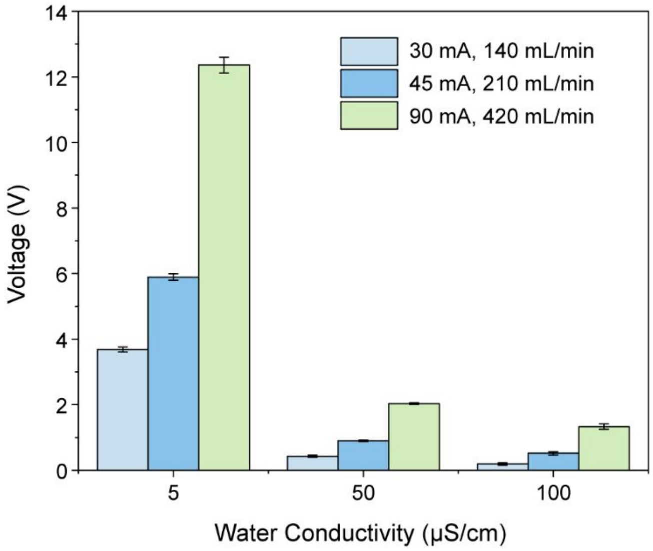

Figure S21. System voltage measured under various flow rates, currents, and water conductivities.

Table S1. Detailed information about the device parameters and data collection of flow cytometry.

Channels	Voltage (V)	Threshold
**FSC-A**	60	2000
**SSC-A**	60	/
**B525-A**	100	/
**B690-A**	600	1000

Table S2. The parameters for energy cost calculation.

Parameter	Unit	Value	Note
Applied Current (I)	A	9×10^{-2}	Experimental result
System voltage (U)	V	1.33	Experimental result
Power (P)	W	1.2×10^{-1}	$P = U \times I$
Flow rate (Q)	mL/min	420	Experimental result
Energy consumption per Liter of water treated (W)	J/L	17.1	$W = \frac{P}{Q} \times 6 \times 10^4$
